# Working in a relational way is everything: Perceptions of power and value in a drug policy-making network

**DOI:** 10.1186/s12961-024-01225-4

**Published:** 2024-10-03

**Authors:** Naomi Zakimi, Martin Bouchard, Alison Ritter, Alissa Greer

**Affiliations:** 1 School of Criminology, Simon Fraser University, Burnaby, BC Canada; 2 Drug Policy Modelling Program, Social Policy Research Centre, University of New South Wales, Sydney, NSW Australia

**Keywords:** Policy network, Social network analysis, Power, Value, Drug policy, policy-making process, Participation

## Abstract

**Background:**

The development of drug policies has been a major focus for policy-makers across North America in light of the ongoing public health emergency caused by the overdose crisis. In this context, the current study examined stakeholders’ experiences and perceptions of power and value in a drug policy-making process in a North American city using qualitative, questionnaire, and social network data.

**Methods:**

We interviewed 18 people who participated in the development of a drug policy proposal between October 2021 and March 2022. They represented different groups and organizations, including government (*n* = 3), people who use drugs-led advocacy organizations (*n* = 5), other drug policy advocacy organizations (*n* = 5), research (*n* = 3) and police (*n* = 2). Most of them identified as men (*n* = 8) and white (*n* = 16), and their ages ranged between 30 and 80 years old (median = 50). Social network analysis questionnaires and semi-structured qualitative interviews were administered via Zoom. Social network data were analysed using igraph in R, and qualitative data were analysed using thematic analysis. The analyses explored perceptions of value and power within a drug policy-making network.

**Results:**

The policy-making network showed that connections could be found across participants from different groups, with government officials being the most central. Qualitative data showed that inclusion in the network and centrality did not necessarily translate into feeling powerful or valued. Many participants were dissatisfied with the process despite having structurally advantageous positions or self-reporting moderately high quantitative value scores. Participants who viewed themselves as more valued acknowledged many process shortcomings, but they also saw it as more balanced or fair than those who felt undervalued.

**Conclusions:**

While participation can make stakeholders and communities feel valued and empowered, our findings highlight that inclusion, position and diversity of connections in a drug policy-making network do not, in and of itself, guarantee these outcomes. Instead, policy-makers must provide transparent terms of reference guidelines and include highly skilled facilitators in policy discussions. This is particularly important in policy processes that involve historical power imbalances in the context of a pressing public health emergency.

**Supplementary Information:**

The online version contains supplementary material available at 10.1186/s12961-024-01225-4.

## Background

In the context of the overdose crisis impacting many cities across North America [1, 2], now widely recognized as a public health emergency [3–6], drug policy has become a growing concern for policy-makers. Several policies have been implemented as a response to the drug overdose crisis, such as drug decriminalization [7, 8], safer supply programs [9] and naloxone distribution [10]. Given the complexity of this public health emergency, understanding how drug policies are created in this context can shed light on how to improve future policy processes. As such, the current study explores a drug policy-making process that took place in a North American city between 2021 and 2022,^1^ focusing not on the content of the policy itself but on the relational process of creating new policies.

The study of drug policy-making is generally concerned with understanding the constellations of institutions and actors involved in the policy-making process, often drawing from policy process frameworks that emphasize the importance of social connections, such as Sabatier’s advocacy coalition framework [11, 12] and policy network theory [13–15]. Within these policy processes, previous research has questioned who gets to participate, what kind of knowledge is more valued and how participation is co-constructed in policy-making [16–20].

There is a breadth of literature exploring the different modes of participation in policy-making processes. While some notions of participation propose a strict definition of the concept, such as deliberative democratic processes (for example, deliberative polling [21]), participation can also be defined more broadly to include the diverse ways in which people are included in policy-making, such as protests and informal working groups [17, 22–26]. In this way, policy participation can be studied as more than a singular and static event with pre-established definitions of participation. Instead, it can be understood as a co-produced and relational process “in the making” ([16], p. 31) [16, 25–27]. This broader approach to understanding participation is the one taken here, which has strong resonance with the policy process under study (a committee process), is sensitive to the specific socio-historical context and is premised on people’s experiences of participation being relational and made within the participatory process.

In seeing drug policy-making as a relational process, it is important to study relationships among participants, which can shape their experiences of participation [28]. Each person comes to participation with a pre-existing network of personal relationships and connections. These relationships can be captured using social network analysis (SNA), a method to study patterns of relationships among social agents [29]. To date, different policy networks have been studied using SNA methods [30–32], such as public health [33], labour [32] and environmental [34] policy networks. In drugs research specifically, most studies using SNA methods have been published in the public health and criminology fields [35]. Some studies have used news articles to map discourse networks of agreement or similar interests [36, 37]. For example, Hilton et al. [37] analysed statements made by UK alcohol policy stakeholders in news articles and mapped a network of agreement across policy actors. Such studies are concerned with the discourse surrounding specific policy debates rather than studying the policy-making process itself. Other studies have used policy documents and interviews to analyse policy networks [38–41]. Weishaar et al. [39, 40] used policy documents, consultation submissions and websites to map a network of European tobacco policy actors, as well as qualitative interviews. Findings provided a nuanced perspective on how actors who opposed smoke-free drug policy connected with each other, forming groups and alliances that were not as clear-cut as was often theorized. SNA studies of drug policy such as this one demonstrate the utility of this method to observe the policy process at the meso-level, providing a bird’s eye view of interactions and underscoring the importance of relationships in policy-making.

However, while it is often assumed that participation should empower participants and allow underrepresented voices to be heard, experiences and perceptions of participation can vary [42]. The power imbalances that exist outside of policy-making processes are rarely challenged, even when different stakeholders and community members are invited to participate, leading to feelings of disappointment and disillusionment when participants feel unable to influence policy [42, 43]. For instance, in drug policy, people who use drugs (PWUD) have reported feeling “tokenized”, unheard and powerless to influence decisions, reproducing existing power relationships and reinforcing their marginalized position [44, 45]. However, scientific knowledge is generally highly valued, as policy-makers aim to create evidence-based policy on the basis of what is perceived to be objective and rational information [45]. As such, how valued and powerful participants feel may vary widely on the basis of the type of relationships among them and the specific context of a policy process.

### Study aims

The current study sought to contribute to the growing literature on drug policy networks and participation by mapping a partial network of stakeholders who participated in a drug policy-making process, and questioning what it means to feel valued and powerful in this context. Specifically, we aim to answer the following research questions: (1) how does a drug policy-making network look from the perspective of stakeholders who participated in the policy-making process? And (2) how are value and power constructed in a drug policy-making network? While there is a common understanding that participation should empower participants and the communities they represent, the current study examines how and who participation empowers and values. We examine these questions from different perspectives to provide a well-rounded understanding of the policy network: relationally through SNA, quantitatively through questionnaires and qualitatively through interviews. Ultimately, the current study can shed light on how to encourage democratic participation in drug policy.

## Methods

### Case study

The current study examined a network of people involved in the development of a new illicit drug policy using social network, quantitative and qualitative data.^2^ While we cannot provide specific information about the specific policy or city where the process took place due to strict confidentiality and ethical restrictions, the process was concerned with developing a proposal for a drug policy that aimed to decrease drug-related harms caused by previous drug policies. This policy was portrayed by government as a helpful step towards solving or addressing the harms associated with the overdose crisis. The city where the process took place has been receptive to implementing progressive liberalization drug policies in recent years compared with other jurisdictions in North America.

At the time of data collection, this policy process had recently concluded, and a policy proposal was submitted to a higher level of government for review. The process was conducted over a period of approximately 3 months. The policy development, organized by local government officials, involved the participation of representatives from different organizations and individuals, including advocacy groups, researchers, police and government officials.

Participation entailed formal meetings (with no public record of the number or duration of these), protests and public demonstrations, media interviews and private discussions between participants. There were no terms of reference for the formal meetings. Public statements made by government officials showed that they were interested in “reaching consensus” among different institutions and communities to shape the policy outcome, describing a traditional process of consultation where government had the final say. However, from interviews and media, participants from advocacy groups believed they would be involved throughout the whole policy process, including decision-making. There is no publicly available information about the specific individuals or government official(s) who wrote the policy proposal; based on our research, the government officials who participated in our study likely had some influence on the contents of the proposal but did not personally write it.

### Recruitment

The research team collected publicly available information about the drug policy, including news articles and the proposal instigators’ meeting minutes, to develop a list of people who may have participated in the process. From this list, we contacted 29 people via email on the basis of availability of contact information and prior working relationships. We also relied on snowball sampling, recruiting individuals named by participants. Members of policy advocacy groups and researchers were more easily recruited than government employees and police officers. However, it was difficult to recruit individuals working for government, especially those who were public figures or so-called political elites [46].

Overall, 18 of the 29 (62%) people contacted agreed to participate; 7 did not respond, and 4 declined our invitation.^3^ We interviewed at least two individuals from each policy group (government *n* = 3, PWUD-led advocacy *n* = 5, drug policy advocacy organizations *n* = 5, research *n* = 3 and police *n* = 2). PWUD-led advocacy actors included people who worked in a PWUD-led policy advocacy group, as well as people with lived experience; however, all of them mentioned also being associated or standing in solidarity with multiple PWUD-led organizations. Drug policy advocacy actors included people who represented the interests of four different non-PWUD-led advocacy groups. All of these groups advocated for the rights of PWUD (and in some cases the rights of other communities), and generally supported drug liberalization and harm reduction policies.

Due to concerns about confidentiality because of the small network to which participants belong and the risk of identification, we report limited information about participants’ demographics, the specific policy process and its location. Most identified as men (*n* = 8) and white (*n* = 16), and age ranged between 30 and 80 years old (median = 50).

### Data collection

Between October 2021 and March 2022, the first author (N.Z.) conducted 17 interview-administered questionnaires and 18 semi-structured interviews via Zoom one-on-one with people who had participated in the policy process.^4^ The interview audio was transcribed by research assistants, and the first author reviewed these for accuracy. To promote confidentiality, participants were invited to review their interview transcript; only three participants reviewed and made minor edits related to anonymization (for example, references to specific relationships) and grammar.

The questionnaires and qualitative interviews were administered consecutively: first an in-screen network questionnaire (approximately 15–30 min), followed by a qualitative semi-structured interview (approximately 30–45 min). For the questionnaire, we asked participants to list their top six “people that [they] interacted with most frequently in the [drug policy] process.” Interactions represented any type of communication, including in-person and online or phone meetings. Then, we asked participants a series of questions related to the overall policy network, such as their own perceived value and power and that of other members. To assess their perceptions of value and decision-making power, we asked the following two questions: “On a scale of 1–10, how valued was your voice or opinion in developing the [drug policy]?” and “On a scale of 1–10, what degree of decision-making power did you have in the [drug policy-making process]?” (Table 4). We defined “value” with a prompt shown on screen, “When you talk, do other people listen and consider your voice or opinion?” and “decision-making power” using “Can you override other people’s decisions?”.

Qualitative interviews facilitated narratives on participants’ experiences in the drug policy-making process. We used a question guide to ensure all topics of interest were covered, but also allowed participants to guide the conversation. Question guide topics included views on the drug policy-making process and network, value and power and the experience and outcome of the policy process. Additionally, the interviewer (N.Z.) wrote memos reflecting on interviews throughout the research process. These memos served to inform the data analysis and findings.

### Data analysis

#### Social network analysis

Network data were analysed using *igraph* [47], an R library used to visualize and analyse graphs and networks. In these networks, each circle represents an ego (someone who participated in our study), and each square represents an alter (someone who was named by a participant but who did not participate themselves).

Three individual-level network measures were calculated for participants: degree and in-degree centrality, and betweenness centrality. Degree centrality measures the number of outgoing and incoming connections for each network member, while in-degree centrality calculates only the number of incoming ties (or nominations) for each member [48, 49]. Betweenness centrality captures the number of times that nodes connect otherwise unconnected nodes, acting as brokers.

Whole network measures were also calculated. Degree centralization measures the extent to which a network revolves around a small number of members, identifying potential leaders (or lack thereof) [50]. Average degree captures the average number of ties each node has in the network [50]. Density is the number of existing connections or ties in a network by the total number of possible ties [49]. Finally, the external–internal (E-I) index examines whether network members tend to connect more with actors within their own group or outside their group [51], indicated by scores where 1 indicates that all members form ties with people from outside their group and −1 shows that all members form within-group ties.

Given that each connection represented communication between the two individuals, any tie between actors implied communication flowing both ways. Therefore, except for in-degree centrality (that is, how many times someone is named by others as a contact in the policy process), we present undirected measures by considering any connection to be mutual (A—B), rather than considering the direction of the relationship (that is, who had named whom, such as A→B, A←B or A←→B).

#### Questionnaire data analysis 

Two questions from the questionnaire were used to assess participants’ self-perceived value and decision-making power in the network. We report raw scores for each participant, on a scale of 1–10, with 1 being no value or decision-making power and 10 being a high degree of value or decision-making power. Additionally, we calculated the average score for each group (government officials, researchers, PWUD-led advocacy, other drug policy advocacy groups and police).

#### Qualitative thematic analysis

Qualitative interview data were initially analysed guided by reflexive thematic analysis [52], which aligns with an interpretive approach [see Additional File 1 for the Consolidated Criteria for Reporting Qualitative Research (COREQ) Checklist]. Data were coded and organized in NVivo [53]. The first and last authors (N.Z. and A.G.) led the qualitative analysis by engaging in in-depth data familiarization, reading all interview transcripts multiple times, developing a coding framework on the basis of the early social network analysis and then conducting two rounds of inductive coding, identifying meaningful patterns in the data, such as “feelings of tokenization” and “police power” (see Additional File 2 for detailed coding tree).

We then referred back to the quantitative and SNA findings to corroborate findings and expand our understanding of the drug policy network. Here, we deviated from a reflexive thematic analytic approach [52], as we more deductively examined the different perspectives from each dataset together to explain perceptions of participation, power and value. Throughout the coding process, we met as a team to discuss possible interpretations of the data, as well as to make sense of similarities and differences. We identified two topic areas that were used to organize and bring together all the findings: (1) perceptions of power and (2) perceptions of value (Table 1). From here, we revisited and recoded the qualitative dataset to develop relevant themes.

**Table 1:** List of topic domains developed from qualitative analysis with example quote

Topic domains
1. Questioning decision-making power in drug policy
1.1.* Separation and lack of information as power*
1.2.*“To be fair…”: perspectives of power from the top*
2. Perceptions of value in the policy-making process
2.1 *Finding value in the process and the outcome*
2.2 *Relationships matter: the value of connections*
2.3 *Competing for value: a finite resource?*

## Results

### Describing the policy network

**Fig. 1:**
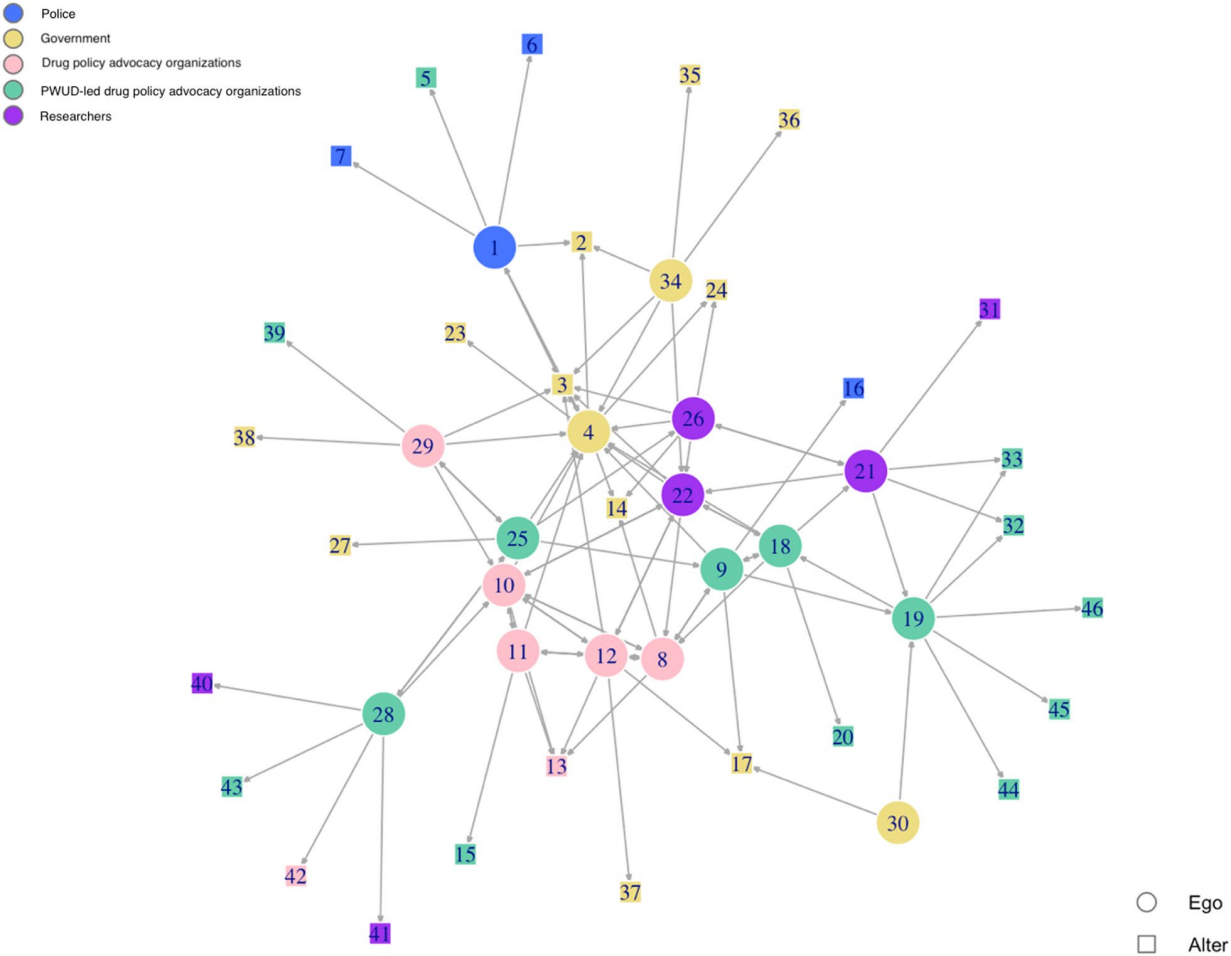
Drug policy-making network (17 egos and 30 alters)

Figure 1 depicts the network of participants’ self-nominated contacts with whom they interacted most frequently throughout the development of the drug policy. Circles represent participants in the study, and squares are alters (individuals named by participants who did not participate in the study). We can see that there was a combination of connections within and across groups. Connections were mixed among government, drug policy advocacy groups, PWUD-led advocates and researchers in the middle of the network; however, police (in blue) were located towards the periphery, suggesting that most participants did not interact with them frequently. The network visual provides an imperfect but still informative way to get an overall snapshot of the drug policy-making network.

The network metrics (Table 2) support the network map in showing that each member connected to only a few others around them. On average, each actor had 3.7 connections, and the density score shows that only 8% of all possible connections were present. In terms of centralization, the network was largely decentralized and did not revolve around one or a few individuals. The external–internal (E-I) index was close to zero (0.06), indicating an almost perfect balance of within-group and between-group connections with a slight preference to connect with members of other groups. This E-I index score means that people’s connections were diverse, communicating with others in their own group as much as across groups.

**Table 2:** Whole network measures for sampled and full network

Whole network measures	Sampled network	Full network
Number of nodes	36	46
Number of edges	63	85
Average degree	3.50	3.70
Density	0.10	0.08
Degree centralization	0.27	0.25
E-I index	−0.05	0.06

Table 3 shows the five participants who scored the highest in three individual-level measures: degree centrality, in-degree centrality and betweenness centrality. Based on this analysis, Participant 4 was the best positioned in structural terms. This person, a government official, had the most overall connections with other network members (degree centrality = 0.33) and was also the one most often named as a connection by other participants (in-degree centrality = 0.22). They were also the main broker (betweenness centrality = 0.36), connecting otherwise unconnected individuals.

**Table 3:** Individual-level network measures of the five highest scoring participants

Measure	Highest-scoring participants	Group	Score
Degree centrality	1. Participant 4	Government	0.33
	1. Participant 22	Research	0.22
	1. Participant 19	PWUD-led AG	0.20
	1. Participants 8, 10 and 12	AG, AG and AG	0.18
In-degree centrality	1. Participant 4	Government	0.22
	1. Participant 3	Government	0.16
	1. Participants 22, 8 and 10	Research, AG and AG	0.13
Betweenness centrality	1. Participant 4	Government	0.36
	1. Participant 19	PWUD-led AG	0.18
	1. Participants 9 and 28	PWUD-led AG and PWUD-led AG	0.17
	1. Participant 25	PWUD-led AG	0.15

*AG* advocacy group

In practice, a central or bridging node in the network does not necessarily equate to decision-making power. Actual brokerage power also depends on the social proximity of the parties involved, as well as on the content or substance of the interactions – it could be that a broker sits in the middle of otherwise weak ties that do not share as much as they would with strong ties [54]. Qualitative data helped expand the network results by examining interactions, and perceptions of power and value.

### Questioning decision-making power in drug policy

#### Separation and lack of information as powerlessness 

When we asked participants to score their own degree of decision-making power, most participants reported having a low degree of power, scoring themselves 5/10 or below (Table 4). Only three people ranked their power as higher than 5/10: Participant 4, a government official (6/10); Participant 1, a police officer (6/10); and Participant 10, a drug policy advocacy group member (7/10). The prevailing sense of powerlessness among participants, as reflected in their questionnaire scores, was consistent with qualitative explanations of feeling separated and silenced by others in the network. This disconnection was evident in quotes referring to limited access at the so-called kids’ table and being barred from higher-level conversations happening behind the scenes, so to speak, where decisions were made: “*we could never go to that table, you know? I said I didn’t want us to be at the kids’ table, I wanted to be at the decision-making table. We could never go there*” (Participant 9, PWUD-led advocacy).

**Table 4:** Value and decision-making power scores (*n* = 16)

Participant	Group	Self-perception of value	Self-perception of decision-making power	Degree centrality	In-degree centrality	Betweenness centrality
4	Government (*n* = 2)	9.0	6.0	0.33	0.22	0.36
34		6.5	3.0	0.13	0.0	0.09
8	Drug policy advocacy (*n* = 3)	7.0	3.0	0.18	0.13	0.05
10		7.0	7.0	0.18	0.13	0.12
11		6.0	2.0	0.13	0.07	0.05
12		7.0	1.0	0.18	0.09	0.08
29		2.0	1.0	0.13	0.02	0.09
1	Police (*n* = 1)	7.0	6.0	0.13	0.13	0.02
9	PWUD-led policy advocacy (*n* = 5)	1.0	1.0	0.16	0.07	0.17
18		7.0	3.0	0.16	0.07	0.11
19		6.0	1.5	0.20	0.07	0.18
25		3.0	1.0	0.13	0.04	0.15
28		7.0	3.0	0.13	0.02	0.17
21	Research (*n* = 3)	7.5	3.5	0.16	0.04	0.11
22		2.0	1.0	0.20	0.13	0.10
26		4.0	1.0	0.16	0.04	0.05

There was a sense of disconnect from more powerful individuals, who were thought to have a separate network where additional conversations and final decisions were made. Lacking access to this group of powerful individuals and to information about the details of the policy design during the policy-making process resulted in a sense of powerlessness:

*They had separate tables and collaborations, so they had one table with people with lived experience, but at the end of the decision making, whoever makes those final decisions, we weren’t involved in that, in those conversations. If we’re going to be at the table then put us in the decision-making process to at least not be blind-sided after the fact of – “this is what we’re doing, and we did consult with people with lived and living experience.” Yeah, big deal, you consulted but you didn’t listen.* (Participant 25, PWUD-led advocacy).

There was a sense of disappointment from the expectation that they would be able to participate in making decisions, a process that was seen as separate and hard to reach. Many participants believed that the government officials who organized the policy-making process, such as Participants 4 or 34, excluded many of the participants we interviewed.

Adding to the feeling of powerlessness through the separation of groups, many participants felt that not enough information was provided about the role of each participant and where power lay. This uncertainty contributed to a sense of powerlessness as participants were unsure where to direct their efforts and resources:

*We had questions about who ultimately decides and where do our comments go and all that. It wasn’t very clear. It was clear that we were at the bottom of a hierarchy. And this is the set of meetings where we weren’t able to access [specific information about policy design], we asked for [it]. We weren’t able to get a kind of clear role on governance, like they were not very transparent about how the decision-making process worked and all that stuff.* (Participant 9, PWUD-led advocacy).

Furthermore, lack of communication and an inability to reach individuals with more decision-making power shaped participants’ views about the process:

*There was a lot of talking together with each other [participant’s colleagues], you know, sharing our same experience and trying to put together pieces also of what’s happening at this higher level because we don’t – I don’t know, we were sort of totally left in the dark.* (Participant 34, government official).

*[Police] probably has the greatest amount of influence but maybe some of the smallest numbers of actual meetings about the [drug policy] because we weren’t able to get meetings with [police]. They are pretty closed off to having meetings because they already have it in their head what [policy design] they want and what they really want to agree to.* (Participant 8, drug policy advocate).

There was a perceived lack of transparency and communication ultimately produced a sense of secrecy and frustration among participants, who alluded to there being “higher-level” meetings and processes in which they could not participate.^5^ One participant explained this sense of separation and secrecy:

*This is a network of people who are advocating and pushing and trying to advance and make changes, but I don’t see them being very connected and integrated into actual government structures and power. So, I think that everybody, like the network, is not a drug policymaking network, it’s a drug policy influencing network.* (Participant 22, researcher).

Some participants did not see themselves as participating in the drug policy-making network, but as a separate network “pushing” to change policy and influence an elusive “higher-level” network. This distinction perceived by participants is important; the network map may suggest that they had access to government officials and some of them had advantageous positions, but they felt separated and isolated from those most powerful. For many, having power would have meant belonging to this other network they were excluded from.^6^

#### “To be fair…”: perspectives of power from the top 

In contrast to most participants who rated themselves as having little to no power, the three participants who rated themselves as having slightly more power (above 5/10) perceived the process differently (Table 4). Overall, they saw the process as more balanced or “neutral” than other participants who did not feel they had power, but also acknowledged and were critical of its shortcomings.

For instance, Participant 4 was in a gatekeeping position; they held the power to provide access or connect different parts of the network (for example, police and PWUD) who would otherwise be disconnected, and had the opportunity (or responsibility) of brokering between these different “sides”. This position may explain their “balanced” or neutral perspective. While they acknowledged some of the shortcomings of the process, they regularly framed the overall experience as positive given the circumstances: “*I think in an imperfect situation under a really tight timeline the [local government] did do a reasonable job at that [connecting with people who use drugs] and there weren’t any big gaps in terms of stakeholders*” (Participant 4, government official).

Participant 10 had a similar reading of the process, which they thought had happened in a “*less-than-perfect situation*”. As a drug policy advocate, they believed that this policy change, however small, was a step in the right direction:

*Those are calculations that do have to be political at times and pragmatic. Do you get what you want, or do you hold out for better? There’s a risk either way and that played out really quite – it wasn’t pretty. [Advocacy group] was really pissed off. The [government official] sort of, like, “Jesus, I’ve gone way out on a limb here and I’m getting shit from all sides now”. Is this the best [policy design] in the world? It might be. It’s a low bar – there’s a lot of really shitty [policy designs] out there.* (Participant 10, drug policy advocate).

Compared with other network members, Participant 10 was also well-connected, scoring in the top five highest in-degree and degree centrality measures (Table 4). They were mentioned as frequent contacts by others in the network, and overall, they had more connections than others (either outgoing or incoming). Many of these network ties may have existed for a long time prior to this process and should continue to exist after it; thus, being pragmatic, so to speak, can help foster some of the relationships that are key in advocating for change. This position could be particularly important for this participant who works at a national-level drug policy advocacy organization, which may require coordinating a variety of different local groups to push towards a common goal.

Compared with Participant 4 and 10, Participant 1 (police officer) was slightly more critical of the process and decision-making. They believed police officers’ concerns were not considered, but also thought that the process had been “well-intended” and that some of its shortcomings were understandable given the circumstances: “*I’ve talked about it got politicized and kind of ideologically driven, but also too, with that time constraint and the backdrop of politics that, you know, we were running ahead at full steam*” (Participant 1, police officer). Furthermore, despite believing their concerns were not considered in designing the policy, this participant believed that, relative to other stakeholders, they had more power and opportunity to participate in higher-level decision-making tables:

*… the voice of police has power… we actually have a lot more power than a lot of other stakeholders […] It comes down to police, health, and government.* (Participant 1, police officer).

Knowing that police officers believed they were more powerful than other stakeholders provides a better understanding of their network position and the dynamics. While this officer and others were hard to reach (both in the network and for this study), their isolation was not due to lack of power. Instead, it may signal their participation in other, higher-level networks of government (as pointed out by Participant 1).

Across these three participants (#1, #4 and #10), their perspectives reflect a sense of control and power throughout the process in different ways. By connecting with people from different organizations, maintaining a variety of relationships with network members, organizing the policy process itself and having access to higher levels of government, these participants were arguably some of the most important network members in terms of influence in the final policy design.

### Perceptions of value in the policy-making process

When participants were asked to rate how much they thought their voices had been valued in the policy-making process, most (11 out of 16) reported a score above 5/10, indicating they felt they had been more than “somewhat valued” (Table 4). These participants represented all five groups (drug policy advocacy, PWUD-led policy advocacy, researchers, police and government). Participant 4, who was in a structurally advantageous position in the network by acting as a broker and having many connections, rated themselves 9/10 in terms of value – the highest rating in the sample.

In framing perceptions of value, we accounted for the way in which underlying power structures influence participants’ experiences within the network. As evidenced in the previous topic domain, police were seen by PWUD advocates and researchers as having had outsized power to influence the final decision-making: “*They [police] have tons of power*” (Participant 25, PWUD-led advocacy). Participant 1, a police officer, also believed they had power in the network (topic domain 1), and the final policy proposal was indeed influenced by the opinion of the local police department. The power imbalance between PWUD advocates and police or government institutions must be taken into account when interpreting participants’ experiences and expectations.

#### Finding value in the process and the outcome 

While most participants (*n* = 11) rated their value as above 5/10 (Table 4), the sentiment described in the qualitative interviews was mixed. The idea of feeling heard relied on being invited to participate in the policy process, the quality of the relationships with other stakeholders and the final outcome. Participants appreciated being included in the network and being asked to share their perspectives, which may be reflected in the relatively high or moderate ratings of value and in the network data. The network map shows that participants communicated with a variety of stakeholders across different groups.

However, value was not only a question of inclusion and participation but also of knowing where and how opinions were taken up in decision-making. For many of these participants who believed they had been at least somewhat valued, there was a sense of feeling initially heard, but later dismissed or “watered down” when learning about the final policy design.

*You get a lot of like “Wow, your information is great! This is amazing. Oh my God, we’re so happy to be involved in this process”, and so much more pleasantries. But the actual actions that happen almost always go in the direction of the safest route, the easiest route, the most comfortable, which is usually towards policing and usually towards the medical system. So it [drug policy] doesn’t actually take the opinions of people who use drugs […] We water them down and push them through the medical system or the criminal system, and then get a really shoddy version of it.* (Participant 18, PWUD-led organization advocate).

Participant 12 expanded on this idea that their perspectives would not be considered or would be considered only in part. They suggested that external political considerations explained why some perspectives and suggestions were not fully considered in the policy design, adding to Participant 18’s concerns about the government doing what is “most comfortable”:

*There were a lot of usual political, you know, professing: “well, of course we want to hear about like frontline lived experience of people who use drugs. But not if it’s going to push us as decision mak[ers] – as [city] into a terrain where we’re requesting something that we don’t feel politically comfortable doing.” That was then kind of devalued. (Participant 12, drug policy advocate).*

Researchers felt similarly about the way their own contributions were not considered in full by decision-makers. They were confused when government officials requested their expertise and data but did not use this information to draw conclusions that they believed were not fully supported by the data. In their view, their data were not used appropriately by decision-makers:

*[Decision-makers] actually made the mistake of including those data and those stated limitations in their report, and then completely ignoring that data and producing [a different decision] than what our data suggested… [then] kind of portrayed it as if they relied on this data from researchers.* (Participant 21, researcher).

Overall, perceptions of value were impacted by the way in which the policy-making process unfolded and by the final outcome. There was a sense of distrust of government institutions both during this process and after. While there was initial hope and assumption that participants’ opinions would be considered from beginning to end and that the policy would “meaningfully engage” with community, participants’ expectations were not met.

#### Relationships matter: the value of connections 

The frustrations surrounding feeling devalued provide important context for the network data. Some participants were in structurally advantageous positions, particularly in terms of being able to connect people to others in the network (betweenness), but did not feel as though they were valued. Interestingly, Participant 9, a PWUD-led group advocate, was one of the top-five brokers (betweenness = 0.17; Table 3) who named a high-ranking police officer in their network of most frequent contacts. However, they had the lowest rating of self-perceived value and power (1/10; Table 4). In the qualitative interview, Participant 9 clarified their relationship with the high-ranking police officer:

*It was a special [redacted for confidentiality] meeting and that’s where [police officer] lectured to us about what was politically possible and all that stuff. […] We also had an initial meeting before there was even a process, I guess in late [year] [government official] called a meeting of like a handful of people and I talked to [police officer] then. […]Everybody’s very nice [at these meetings], right? Everyone’s like “oh, I’m so glad you came and said these things, it’s really important to listen to lived experience, blah blah blah.” But that’s how people do politics right now.* (Participant 9, PWUD-led group advocate).

This quote illustrates a key point: frequent contact, even with high-ranking individuals, did not necessarily translate into feeling valued or creating meaningful relationships, even though this participant appeared to be in an advantageous brokerage position. Contact in formal policy spaces was not sufficient to feel heard and valued in this case.

Moreover, the level of involvement in the policy-making process may have been different for each participant. For Participant 9, these interactions in formal meetings with Participant 16 (police officer) were frequent enough to be considered in their list of top six contacts, but they did not feel truly heard and were “lectured” instead. Thus, the quality of the connection and interactions appears to be important, and it may explain the discrepancy between the high betweenness centrality and the extremely low value (and power) score for someone such as Participant 9.

The importance of relationships and the quality of the connection was reinforced through the qualitative data. A government official who felt as though their own expertise and that of marginalized communities had not been valued explained the importance of relationships in policy-making:

*One of the things I’ve learned from working with Indigenous community is when you mess something up with a relationship from the outset you are fighting an uphill battle all the way. Relationships from the outset are everything, working in a relational way is everything, and I think we have a lot of really great lessons learned* (Participant 34, government official).

Underlying this topic domain is the idea that a policy process where participants feel valued is one where “evidence” (however that is defined, for example, experience, opinion, quantitative data, qualitative data, etc.) is used to arrive at what participants view as the most logical and fair conclusion. However, feeling valued becomes challenging when individual relationships among participants do not feel genuine. The context in which these relationships exist, often one where there is a significant power imbalance between people with lived experience and official government institutions, shapes experiences of value. The network data show that participants communicated with each other, even across groups that would have had different perspectives (for example, police and PWUD). However, communication and being heard, so to speak, in a diverse policy network are not sufficient to make participants feel valued. The quality of relationships and the access to information about how the outcome will be decided are also important.

#### Competing for value: a finite resource?

Overall, there was a sense of competition for value among participants. For instance, many participants believed that police officers were more valued by government than the advocates and researchers involved: “*in general, it [policy-making network] really overvalued law enforcement’s opinion and it did not adequately include people with lived and living experience*” (Participant 26, researcher). The idea of “overvaluing” proposes that there are certain perspectives that can receive too much or excessive value beyond what they should receive. From this perspective, value for those representing the interests of PWUD was assessed in comparison or opposition to that of police officers:

*The police force has always had a very strong voice in drug policy decision making whether their views are evidence-based or not. So, I would characterize [the network] again as being aspirational, a bit overreaching? And a little bit too deferential to the opinions of the enforcement community*. (Participant 21, researcher).

The degree of value assigned to each group was tied to participants’ own positioning/group, with no consensus across all participants about who was over- or undervalued.

Many participants who represented the interests of PWUD compared their value with that of police officers, while police officers believed that PWUD advocates were overvalued. Participant 6, a police officer, illustrated their frustration around the types of knowledge that were most valued in the policy-making process:

*On one hand, I think that lived and living experiences of everybody at the table – our First Nations and all that was critical, health authorities who were there. But I also think at times not everyone’s lived or living experiences was applied equally. It was really more so that the persons who use drugs whose really voice in that one was loudest. (Participant 6, police officer).*

Even though most participants, regardless of group, rated their own value in the process as relatively high (above 5/10; Table 4), this did not translate into feeling valued enough compared with other groups. Comparing the value of different voices, so to speak, implied the perception that some were louder than others and, therefore, muted other participants. By competing, so to speak, value is understood as a finite resource that must be distributed among different groups.

This theme raises questions around how to frame value in policy-making. In this case, differing expectations of what participation entailed may have led to a sense of competition to be heard by policy-makers. Framing value as a finite resource in this context may also explain the discrepancy between most participants’ value scores and the qualitative description of policy-makers as having valued some voices over others.

## Discussion

The current study aimed to map and describe a network of interactions among stakeholders who participated in the design of a new drug policy working in the context of a public health emergency in North America, and their perceptions of power and value within the process. Our findings show that feeling valued goes beyond within-network relationships and requires an understanding of relationship quality outside the policy network and that network data alone may not reveal the most powerful participants. We discuss each of these points in turn, as well as their implications.

First, representation or inclusion in the policy network did not translate into feeling valued or powerful. Consistent with previous research that stresses the importance of fostering quality connections in drug policy processes [55], relationship quality, as opposed to mere communication, was important. For some participants, relationships with other network members did not feel genuine or useful due to the context outside of this specific network. Historical relationships/ties that existed outside of the policy sphere influenced how people perceived the network and their own value and power, regardless of network position. Similar to previous research on European smoke-free policy [39], groups that had conflicting relationships outside of this network, such as police and drug policy advocates (or health organizations and tobacco manufacturers), felt divided and disconnected from each other. Specific to this study, there was a pre-existing negative and imbalanced relationship between some stakeholders, particularly between police officers and people who use drugs, that offer important context to these feelings of disconnect and lack of genuine relationships. However, in the current study, this division or disconnect was not clearly shown in the network data but rather in the qualitative perceptions of participants for whom these pre-existing relationships fueled the discontent and competition for being heard, so to speak, or valued from both sides.

In this case, avoiding conflict altogether may not have been possible, but it does not necessarily signify failed participation. For Young [56], conflictual relationships must be acknowledged in such processes by unmasking the differences instead of “pretend[ing] to have common interests” (p. 44) as some formal traditions of democratic participation would suggest is best. Differences in opinion and experience can strengthen democratic processes by “slowing them down” and disrupting notions of what knowledge counts [16]. Fostering relationships is therefore an important part of feeling heard and valued beyond the policy outcome. Failing to achieve full consensus can fuel further democratic activities [16, 17, 23, 57].

A second important finding is that, although network data suggested that some participants may have been strategically positioned to influence policy due to their high betweenness and degree centrality [48, 49], network position and relationships alone did not determine power; qualitative interviews revealed a clear perception of hierarchy with government officials and police at the top. Our findings suggest that actors in peripheral or hard-to-reach positions in a policy network, from the perspective of “lower ranking” or less powerful policy actors, being “hidden” could be a sign of power or of belonging to a separate policy network (as pointed out by some participants) that we were not able to capture. Alternatively, many participants may be in seemingly advantageous positions but connected through weak ties, meaning they do not share as much information and closeness as they would with their strong ties or relationships [54]. While the position of police as the most powerful participant could not be confirmed by the network data, these qualitative perceptions alone are important in understanding how notions of participation were constructed and shaped throughout the process. The “hidden” or unreachable description of certain policy actors contributed to this understanding of participation as disingenuous and devalued by many participants.

This study is not without limitations. Given that this is one of the first attempts at mapping a drug policy-making network by interviewing network members and administering social network questionnaires (as opposed to using pre-existing policy documents), the limitations of the current study can help inform future similar research. Importantly, there was no publicly available and detailed information about how decisions were made within the network, including the weight given to each stakeholder’s perspective. While this lack of information may be a limitation in many cases where the objective is to find what really happened, the current study explored how participants perceived and constructed the network and policy-making process, choosing not to prioritize any one perspective as the absolute truth.

Furthermore, we were unable to access or interview some network members, especially higher-ranking policy actors. More time for the researchers to develop relationships and trust with policy actors may be needed for future work. Relatedly, there was no pre-existing list of individual policy actors who participated in this policy process, making it difficult to establish a network boundary. Instead, we relied on publicly available information and snowball sampling. Furthermore, views of drug policy advocates, both from PWUD-led organizations and other drug policy advocacy groups, were overrepresented in the network and qualitative data. As such, strong conclusions about the perceptions and involvement of high-ranking government and police officials cannot be drawn from the data presented here. Ultimately, our findings cannot be generalized to previous or future policy-making processes; however, they may be transferable and informative to other drug policy-making processes, providing a lens or framework through which to understand participation, value and power beyond the content of the policy itself.

There are also limitations presented by our confidentiality agreement with participants. The inability to mention the city and policy at the heart of this policy-making process may have prevented us from analysing context-specific issues around individual participants and groups, as well as our ability to generalize or draw stronger conclusions about transferability. However, we believe that this compromise is necessary when studying many drug policy-making processes to access hard-to-reach populations, such as political elites in public-facing government roles and high-ranking positions in “highly politicised and contested policy domains” as drug policy tends to be [46]. Offering confidentiality can lay the groundwork for building rapport with a population that is generally hesitant to participate in research studies.

Two main policy implications stem from the current study. First, participation processes in drug policy-making would benefit from having clear guidelines and expectations on what participation entails, including how and who will make final policy decisions [58–61]. Such guidelines may promote trust among participants and the public, as well as accountability among policy-makers. In doing this, drug policy-makers should consider issues of participants’ social positioning, ensuring those without former policy-making experience have an opportunity to clarify expectations for themselves and others “at the table”.

Second, the role of a highly skilled facilitator(s) cannot be understated. Findings show that most stakeholders did not feel as though their perspectives were genuinely valued and considered in the policy-making process. A facilitator can mediate discussions and make participants feel heard and valued beyond the policy outcome, thus impacting how participation in the policy process may be perceived and constructed [62, 63]. As our findings highlighted, participants often felt a lack of genuineness in the relationships established in the policy-making network. Many felt tokenized or dismissed. Given the historical power imbalances and conflict embedded in drug policy-making and the pressing nature of the overdose crisis in many North American jurisdictions (such as the one in the current study), facilitators must have the ability to mediate interactions among participations with opposing perspectives and encourage them to see each other as more than the role or organization they represent, emphasizing the importance of genuine relationships in policy-making.

## Conclusions

Negative experiences in the policy-making process may lead people to disengage and refuse to participate in future endeavours. In the context of the overdose crisis that has become a public health emergency across North America, ensuring that drug policy-making processes engage with a variety of perspectives, including that of people most affected by such policies, is key. Therefore, drug policy-making processes must be meaningfully designed and properly mediated to promote power-sharing and make participants feel valued, emphasizing the importance of communication and relationships.

## Supplementary Information

Additional file 1. Consolidated Criteria for Reporting Qualitative Research (COREQ) Checklist.

Additional file 2. Coding tree.

## Data Availability

The datasets generated and/or analysed during the current study are not publicly available due to confidentiality and ethical restrictions but may be available from the corresponding author on reasonable request.

## Footnotes

1 Due to strict confidentiality restrictions, we are not able to disclose the specific policy in question or the city where the process took place. We provided as much detail as possible in the methods section.

2 The analysis was not pre-registered and, as such, should be considered exploratory in nature.

3 While only 49 participants were potentially available for contact at the start of the study, the network data collection process allowed us to see that up to 83 people may have had a role in the policy process from start to finish. Ultimately our network only captured a portion of the drug policy-making process as a whole, anywhere from 22% (if the full list of participants is indeed 83) to 37% (if we use the 49 names publicly available).

4 One participant agreed to participate in the semi-structured interview but did not answer the questionnaire, as they saw themselves only peripherally connected to this specific network, having had only a few interactions within it.

5 There are very little data available about who participated in the process and where and when meetings took place. It is not possible for us to expand on which meetings participants were left out of and when; however, the current analysis is concerned with their perspectives and feelings throughout the process.

6 There are very little data available about who participated in the process and where and when meetings took place. It is not possible for us to expand on which meetings participants were left out of and when; however, the current analysis is concerned with their perspectives and feelings throughout the process.
