# Supplementary material for: Working in a relational way is everything: Perceptions of power and value in a drug policy-making network
Source: Health Res Policy Syst. 2024 Oct 3;22:139. doi: 10.1186/s12961-024-01225-4 (PMC11448052; doi:10.1186/s12961-024-01225-4)
Supplement: Supplementary file 1 — Additional file 1. Consolidated Criteria for Reporting Qualitative Research (COREQ) Checklist. [file 12961_2024_1225_MOESM1_ESM.docx]

**Additional File 1**

**Consolidated Criteria for Reporting Qualitative Research (COREQ) Checklist**

**Developed by**: Tong, A., Sainsbury, P., & Craig, J. (2007). Consolidated criteria for reporting qualitative research (COREQ): a 32-item checklist for interviews and focus groups. *International Journal for Quality in Health Care, 19*(6), 349-357.

**Manuscript**: *Working in a relational way is everything": Perceptions of power and value in a drug policymaking network.*

| **ITEM** | **GUIDE QUESTIONS/DESCRIPTION** | **REPORTED ON PAGE # / ANSWERS** |
| --- | --- | --- |
| **Domain 1: Research team and reﬂexivity** | | |
| *Personal Characteristics* | | |
| 1. Interviewer/facilitator | Which author/s conducted the interview or focus group? | The first author (NZ) conducted all interviews |
| 2. Credentials | What were the researcher’s credentials? E.g. PhD, MD | The author (NZ) has a master’s degree in Criminology and was a PhD student at the time of the study. |
| 3. Occupation | What was their occupation at the time of the study? | The first author (NZ) was a PhD student and a research assistant at the time of the study. Study design, data collection, analysis, and reporting were all done in collaboration with the three other authors, who all have doctorate degrees and are professors. The second author (MB) is a Professor and the Director of the School of Criminology at Simon Fraser University in Canada. The third author (AR) is a Professor and Director of the Drug Policy Modelling Program (DPMP) at the University of New South Wales in Australia. The last author (AG) is an Assistant Professor at the School of Criminology at Simon Fraser University in Canada. |
| 4. Gender | Was the researcher male or female? | The first author who collected the data identifies as female. |
| 5. Experience and training | What experience or training did the researcher have? | The first author had experience conducting semi-structured qualitative interviews for other research studies in the previous three years, as well as using thematic analysis and using social network analysis to analyze a variety of qualitative and quantitative data.  Study design, data collection, analysis, and reporting were all done in collaboration with the three other authors, who all have doctorate degrees and are professors with extensive experience conducting research about drug policy and participating or contributing their expertise to local drug policy. The second author (MB) also has broad experience using social network analysis, and teaches a graduate-level course about this method. |
| *Relationship with participants* | | |
| 6. Relationship established | Was a relationship established prior to study commencement? | The first author conducted all the interviews and had no prior relationship with any participants, but two researchers (AG, MB) had prior professional relationships with some participants. Having no prior relationship with the participants and relatively little power in the drug policymaking field may have facilitated frank discussions about the topic and allowed for more openness than if the interviews had been conducted by a researcher with extensive experience in drug policy research and participation. |
| 7. Participant knowledge of the interviewer | What did the participants know about the researcher? e.g. personal goals, reasons for doing the research | All participants were aware of the research objectives and the research team’s personal goals. The first author who conducted the interviews explained to participants that the main objective was to better understand the perspectives of people who participated in a drug policymaking process using social network analysis and qualitative interviews. |
| 8. Interviewer characteristics | What characteristics were reported about the interviewer/facilitator? e.g. bias, assumptions, reasons and interests in the research topic | In her role as a PhD student, the first author was in a unique position in the research team to conduct the interviews. She had a genuine interest in learning about drug policy and had no pre-existing knowledge about most participants. She is also an international student from South America, which meant she had been exposed to different approaches to drug policymaking. However, she was guided by the co-authors, who had extensive experience conducting research on drug policy. These factors may have influenced the interviews, such as by making the first author ask questions that may have been taken for granted by a more experienced interviewer. Further, being a student could have influenced how comfortable the interviewer and participants were asking and answering questions. These personal characteristics must be considered when interpreting the study’s findings, both in how they strengthen and limit the analysis. |
| **Domain 2: study design** | | |
| *Theoretical framework* | | |
| 9. Methodological orientation and Theory | What methodological orientation was stated to underpin the study? e.g. grounded theory, discourse analysis, ethnography, phenomenology, content analysis | 9 |
| *Participant selection* | | |
| 10. Sampling | How were participants selected? e.g. purposive, convenience, consecutive, snowball | 5-6 |
| 11. Method of approach | How were participants approached? e.g. face-to-face, telephone, mail, email | 5-6 |
| 12. Sample size | How many participants were in the study? | 6 |
| 13. Non-participation | How many people refused to participate or dropped out? Reasons? | 6 |
| *Setting* | | |
| 14. Setting of data collection | Where was the data collected? e.g. home, clinic, workplace | 6 |
| 15. Presence of non-participants | Was anyone else present besides the participants and researchers? | 6 |
| 16. Description of sample | What are the important characteristics of the sample? e.g. demographic data, date | 6 |
| *Data collection* |  |  |
| 17. Interview guide | Were questions, prompts, guides provided by the authors? Was it pilot tested? | The first author practiced conducting the interview with another research assistant and received training from the last author on interview techniques (AG). |
| 18. Repeat interviews | Were repeat interviews carried out? If yes, how many? | No repeat interviews were carried out. |
| 19. Audio/visual recording | Did the research use audio or visual recording to collect the data? | 7 |
| 20. Field notes | Were ﬁeld notes made during and/or after the interview or focus group? | 7 |
| 21. Duration | What was the duration of the interviews or focus group? | 7 |
| 22. Data saturation | Was data saturation discussed? | In alignment with our qualitative analytic method, a sample size of 18 was justified based on several considerations (Clarke & Braun, 2022) (see Braun and Clarke (2021) for a discussion on the utility of data saturation as a concept to justify the sample size). The decision to stop conducting interviews often goes beyond the concept of “data saturation”, which lends itself to multiple interpretations; sample size is often pragmatic and based on implicit academic rules, funding and time limitations, and the richness of the data (Clarke & Braun, 2022). In the current study, the combination of participants we interviewed, with highly unique and specific experiences, and the topic of interest helped us determine that a sample size of 18 provided a rare and rich insight into a drug policy process (Malterud et al., 2016) despite the difficulty posed by time and resource limitations. |
| 23. Transcripts returned | Were transcripts returned to participants for comment and/or correction? | 7 |
| **Domain 3: analysis and ﬁndings** | | |
| *Data analysis* | | |
| 24. Number of data coders | How many data coders coded the data? | 9 |
| 25. Description of the coding tree | Did authors provide a description of the coding tree? | 9, Figure 1 |
| 26. Derivation of themes | Were themes identiﬁed in advance or derived from the data? | 9 |
| 27. Software | What software, if applicable, was used to manage the data? | 9 |
| 28. Participant checking | Did participants provide feedback on the ﬁndings? | Participants did not provide feedback on the findings. |
| *Reporting* |  |  |
| 29. Quotations presented | Were participant quotations presented to illustrate the themes/ﬁndings? Was each quotation identiﬁed? e.g. participant number | 12-24 |
| 30. Data and ﬁndings consistent | Was there consistency between the data presented and the ﬁndings? | 12-24  The findings reflect patterns of meaning across the experiences and perspectives of participants, analyzed from an interpretive lens |
| 31. Clarity of major themes | Were major themes clearly presented in the ﬁndings? | 12-24  Value and power are clearly divided as major themes/topic domains in the data, with their respective subthemes |
| 32. Clarity of minor themes | Is there a description of diverse cases or discussion of minor themes? | 12-24  We discussed the experiences of those who did not align with the majority throughout the findings. For example, on pp. 14-18, under the theme “To be fair..”, we discuss the unique experiences of the few participants who were in seemingly advantageous/powerful positions. |

**References**

Braun, V., & Clarke, V. (2021). To saturate or not to saturate? Questioning data saturation as a useful concept for thematic analysis and sample-size rationales. *Qualitative Research in Sport, Exercise and Health*, *13*(2), 201-216. https://doi.org/10.1080/2159676X.2019.1704846

Clarke, V., & Braun, V. (2022). *Thematic analysis: A practical guide*. SAGE.

Malterud, K., Siersma, V. D., & Guassora, A. D. (2016). Sample size in qualitative interview studies: guided by information power. *Qualitative Health Research*, *26*(13), 1753-1760.
