# Supplementary figures and images for: Working in a relational way is everything: Perceptions of power and value in a drug policy-making network
Source: Health Res Policy Syst. 2024 Oct 3;22:139. doi: 10.1186/s12961-024-01225-4 (PMC11448052; doi:10.1186/s12961-024-01225-4)

## Additional File 2 Coding Tree

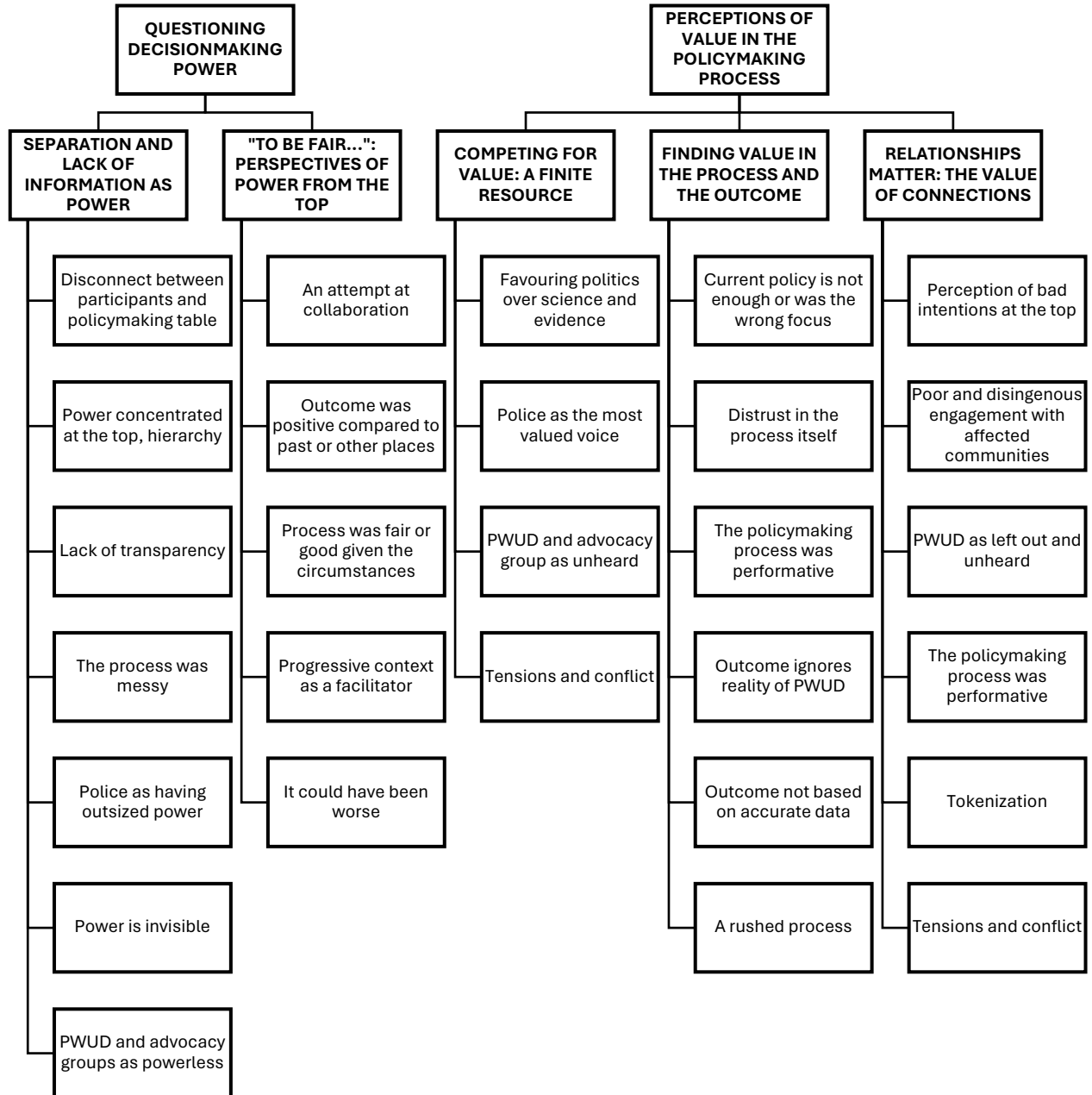

Supplement: Supplementary file 2 — Additional file 2. Coding tree. [file 12961_2024_1225_MOESM2_ESM.pdf]
